# Comparison of Two Antibiotic Combinations for Conservative Treatment of Non-Complicated Appendicitis in Children

**DOI:** 10.3390/medicina62071399

**Published:** 2026-07-19

**Authors:** Mohit Kakar, Georgijs Kulibaba, Zahraa Merchant, Astra Zviedre, Timurs Zurmutai, Lasma Asare, Peteris Tretjakovs, Arnis Engelis, Aigars Petersons

**Affiliations:** 1 Department of Pediatric Surgery, Children’s Clinical University Hospital, Vienibas Gatve, 45, LV-1004 Riga, Latvia; timurs.zurmutai@rsu.lv (T.Z.); arnis.engelis@rsu.lv (A.E.); 2 Department of Pediatric Surgery, Riga Stradins University, Dzirciema Iela, 16, LV-1007 Riga, Latvia; astra.zviedre@rsu.lv (A.Z.); aigars.petersons@rsu.lv (A.P.); 3 Faculty of Medicine, Riga Stradins University, Dzirciema Iela, 16, LV-1007 Riga, Latvia; georgijs.kul@gmail.com (G.K.); merchant.zahraa06@gmail.com (Z.M.); 4 Statistics Unit, Riga Stradins University, Balozu Iela 14 A, LV-1046 Riga, Latvia; lasma.asare@rsu.lv; 5 Department of Human Physiology and Biochemistry, Riga Stradins University, Dzirciema Iela, 16, LV-1007 Riga, Latvia; peteris.tretjakovs@rsu.lv

**Keywords:** appendicitis, children, inflammation, IL6, IL8, MCP1, conservative treatment, antibiotics

## Abstract

*Background and Objectives:* Acute appendicitis is the most common surgical emergency in children. The standard of care has been appendectomy for many years. However, in recent years, studies investigating antibiotic therapy alone have increased rapidly, suggesting that non-operative management may be an effective alternative with a lower risk of complications. In this study, we compared two antibiotic regimens (ceftazidime/metronidazole and ampicillin/metronidazole) to evaluate the effectiveness of two antibacterial strategies. *Materials and Methods:* A total of 41 children aged 7–18 years were enrolled and randomly assigned to one of the two treatment groups, with the follow-up conducted over one year to assess recurrence. For the evaluation of antibiotic effectiveness, inflammation was assessed using leukocyte count, C-reactive protein, and the biomarkers IL-6, IL-8, and MCP-1, which were measured on each of the three days of inpatient antibiotic therapy. *Results:* Analysis of the collected data revealed no statistically significant difference in inflammatory markers between the two treatment groups. *Conclusions:* These findings suggest that both antibiotic regimens offer comparable clinical outcomes and may be used interchangeably in clinical practice.

## 1. Introduction

Appendicitis is the most common surgical emergency in children, with a lifetime risk of 7–8% and a peak incidence in the teenage years [1]. The prevalence of pediatric appendicitis was estimated to be 109 per 100,000. From 1990 to 2021, the incidence of appendicitis in children increased by 0.3% annually [2].

The standard of care has been appendectomy since the first surgery was described in 1886. Non-surgical treatment, such as using antibiotics alone, has only recently begun to be formally compared in trials with appendectomy in adults and in children [3]. Laparoscopic appendectomy for non-perforated appendicitis has a low level of complication risks; however, because it is abdominal surgery requiring general anesthesia, it inevitably exposes children to a risk of complications. Several studies have documented that antibiotic therapy alone is highly successful as an initial treatment for appendicitis and is associated with a more rapid return to normal activity than surgery [4,5].

Minneci et al. (2020) demonstrated that among children with uncomplicated appendicitis, an initial nonoperative management strategy with antibiotics alone had a success rate of 67.1% and, compared with urgent surgery, was associated with statistically significantly fewer disability days at 1 year [6]. A recent randomized controlled non-inferiority trial showed that non-operative management was safe and worked well for pediatric patients with uncomplicated appendicitis; however, the treatment failure and recurrence rate are still important factors to consider in decision-making [7]. The 2020 WSES guidelines recommended intravenous antibiotics, based on evidence that suggests non-operative management may result in shorter hospital stays while being safe and feasible for children with uncomplicated appendicitis [4,8]. Consequently, treatment decision-making is slowly shifting towards individual risk stratification rather than a uniform operative approach [9,10].

Identifying reliable biomarkers to guide treatment decisions remains an important challenge, as inflammatory markers are central to both diagnosis and prognosis in acute uncomplicated appendicitis [11,12]. Leukocyte count and CRP are the most widely used non-specific indicators of inflammation, reflecting innate immune activation and the hepatic acute-phase response, respectively [13,14]. Although individually non-specific, together they aid clinical decision-making and may help monitor treatment response [3,10,12,15,16]. However, conventional markers do not fully capture the complexity of the inflammatory process, prompting growing interest in more specific immunological biomarkers such as IL-6, IL-8, and MCP-1 [17]. IL-6, a pro-inflammatory cytokine central to the acute-phase response, rises earlier than CRP and correlates with disease severity in pediatric appendicitis and has been shown to enhance the diagnostic performance of standard blood tests [13,18,19,20,21]. IL-8 (CXCL8), the principal chemokine driving neutrophil recruitment, reflects the intensity of neutrophil-mediated inflammation within the appendiceal wall and has been linked to more severe disease courses [14,22,23,24,25]. MCP-1 (CCL2), involved in monocyte and macrophage recruitment, is thought to reflect a more sustained phase of inflammation, and may provide complementary information when monitored alongside other cytokines [14,16,25,26,27,28].

The two antibiotic regimens compared in this study, ceftazidime/metronidazole and ampicillin/metronidazole, target both aerobic and anaerobic bacteria associated with intra-abdominal infection but differ in antimicrobial spectrum. Ceftazidime, a third-generation cephalosporin, inhibits bacterial cell wall synthesis and is particularly active against Gram-negative organisms such as Pseudomonas species, whereas ampicillin acts through a similar mechanism but is more effective against Gram-positive organisms with a relatively limited Gram-negative spectrum [29,30]. Metronidazole complements both regimens by disrupting DNA synthesis in anaerobic bacteria commonly involved in appendiceal infection [31]. Recent microbiota analyses conducted at the Children’s Clinical University Hospital in Riga demonstrated that Escherichia coli predominates in cases of uncomplicated appendicitis, whereas Pseudomonas aeruginosa is more frequently associated with complicated disease [32,33]. These findings support the use of third-generation cephalosporins/penicillins, such as ceftazidime/ampicillin in combination with metronidazole for broader Gram-negative coverage, particularly in settings where resistant organisms may be present. Importantly, accumulating evidence suggests that the microbiological profile of acute appendicitis may vary across geographic regions and healthcare settings [34]. Therefore, antibiotic selection should not be universally standardized but instead tailored to local microbiological patterns and antimicrobial susceptibility data. This highlights the importance of region-specific studies, such as ours, in guiding optimized and context-appropriate antibiotic strategies for the management of acute appendicitis.

These differences in antimicrobial spectrum may influence bacterial clearance and the associated inflammatory response, which is relevant when evaluating changes in inflammatory biomarkers during antibiotic therapy. Given these differences in antimicrobial spectrum, the aim of this study was to compare the clinical and inflammatory-biomarker response of children with acute uncomplicated appendicitis treated with ceftazidime/metronidazole versus ampicillin/metronidazole and to evaluate the recurrence rate associated with each regimen over one year of follow-up. The primary endpoint of this study was the change in inflammatory biomarkers (leukocyte count, CRP, IL-6, IL-8, and MCP-1) between hospital admission and the third day of antibiotic therapy, compared between the two antibiotic regimens. The secondary endpoint was the 1-year recurrence rate of appendicitis in each group.

## 2. Materials and Methods

### 2.1. Patient Inclusion Criteria

Patients were included according to the following criteria: age of the patient between 7 and 18 years old, diagnosis of primary acute non-complicated appendicitis, acute appendicitis signs on ultrasound imaging, and Alvarado score ≥ 7. Patients were excluded according to the following criteria: positive pregnancy test, peritonitis, previous abdominal surgery, use of antibiotic drugs in the previous 7 days, inflammatory bowel disease, immunosuppressive conditions, acute complicated appendicitis, and malignancies.

This study was done in accordance with the 1975 Helsinki Declaration as well as independently reviewed and approved. Ethical approval for this study was obtained from the Ethical Committee of Riga Stradins University (Approval No. 6-1/13/2020/31; approval date: 17 December 2020) and Pauls Stradins Clinical University Hospital (Approval No. 290421-21L; approval date: 29 April 2021). This study was also registered in the European Union as a single-center clinical trial (EudraCT No. 2021-001729-38; start date: 2 July 2021). Approval for patient inclusion was granted by Riga Children’s Clinical University Hospital (Approval No. 02-09-488/2021; approval date: 5 July 2021).

The data analyzed in this study were collected between 2 September 2021 and 7 January 2026.

All parents and patients were fully informed about the aim and nature of this study, and they provided written informed consent for participation in the study and its publication.

### 2.2. Study Protocol

Children between 7 and 18 years old, diagnosed with acute uncomplicated appendicitis, were enrolled and randomly assigned to two different treatment groups. One group received ceftazidime/metronidazole; however, the other group received ampicillin/metronidazole. Both regimens were administered for 3 consecutive days. For the inflammation evaluation, blood leukocyte count, serum C-reactive protein, interleukin-6, interleukin-8, and monocyte chemoattractant protein 1 were evaluated every day (3 times overall) during the hospital stay. Day 1 was defined as the day of hospital admission. The follow-up was conducted over the course of a year to assess recurrence. A total of 41 patients were included, with 27 being male and 14 being female. An amount of 19 patients received a combination of ceftazidime/metronidazole, and 22 received an ampicillin/metronidazole combination. Patients were discharged after the hospital stay with orally prescribed amoxicillin/clavulanic acid (Amoxiclav) for the duration of 7 days under supervision of the family physician.

**Figure 1:**
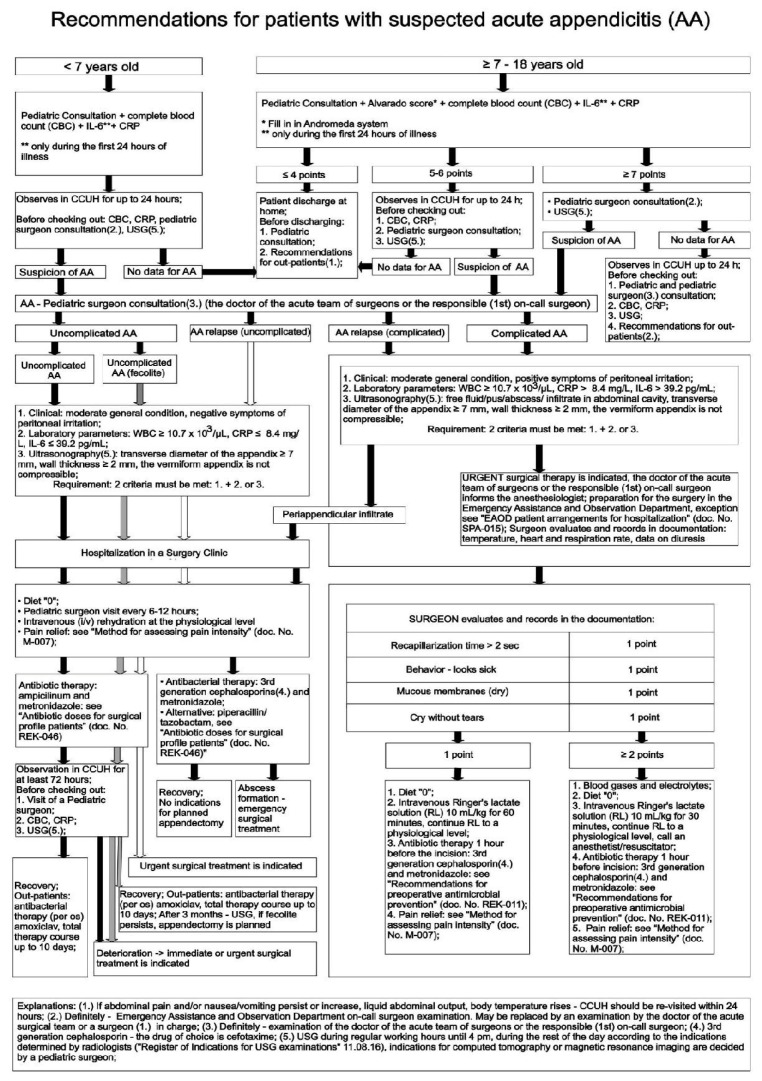
Recommendations for patients with suspected acute appendicitis in Children’s Clinical University Hospital in Riga (REK-052/02).

The antibiotic selection and diagnostic approach for acute appendicitis were based on the Children’s Clinical University Hospital in Riga guidelines (REK-052/02) (Figure 1), which represent the recommendations for patients with suspected acute appendicitis. If a patient received an Alvarado score of seven or more, a surgical consultation was indicated and, if necessary, additional radiological imaging was performed, such as abdominal ultrasound (US) and/or magnetic resonance imaging (MRI).

Recurrence was defined as readmission to the Children’s Clinical University Hospital in Riga within one year following successful conservative management of acute uncomplicated appendicitis, under the assumption that patients experiencing recurrence would present to the same institution. To minimize the risk of unrecorded recurrence occurring elsewhere, all patients were contacted by telephone at the end of the one-year follow-up period by the principal investigator *dr. med.* Mohit Kakar.

### 2.3. Laboratory Analysis

Evaluation of blood leukocyte count and serum C-reactive protein was performed in the laboratory of the Children’s Clinical University Hospital (BKUS) in Riga. Interleukin-6, interleukin-8, and monocyte chemoattractant protein 1 concentrations were measured using the Luminex 200™ (Merck Millipore, Darmstadt, Germany) with a Millipore Milliplex (Merck Millipore, Darmstadt, Germany) magnetic panel in the laboratory of the Department of Human Physiology and Biochemistry of Riga Stradins University. Reference values are measured in picograms per milliliter (pg/mL).

### 2.4. Statistical Analysis

Normality of quantitative data was tested with the Shapiro–Wilk test, and for non-normally distributed data, the results were presented as median and interquartile ranges, with non-parametric tests used for comparison. Mann–Whitney U tests were done for the treatment group comparison. The Friedman test (Bonferroni correction) was used to evaluate treatment effectiveness within one group between several measurements. One-year recurrence rates of acute appendicitis for both groups were compared by calculating the odds ratio. The level of statistical significance was set at *p* < 0.05.

## 3. Results

### 3.1. Characteristics and Distribution of Study Population

This study included 41 children aged between 7 and 18 years who were diagnosed with AuA. Each patient, after signing the agreement, was enrolled and randomly assigned to two different treatment groups. One group received a ceftazidime/metronidazole (Cef/Met) antibiotic combination, while the other group received an ampicillin/metronidazole (Ampi/Met) antibiotic combination. The Cef/Met group was represented by a total of 19 patients, 14 being boys and 5 being girls. The Ampi/Met group was represented by a total of 22 patients, 13 being boys and 9 being girls. Median patient age for the Cef/Met group was 12.95 years and for the Ampi/Met group, 13.18 years (Table 1).

### 3.2. Evaluation and Comparison of Inflammatory Factors Across Antibiotic Groups

Blood and serum samples were obtained from each patient daily for a period of three days. As illustrated in Table 2, median values of analyzed Leu, CRP, IL-6, IL-8, and MCP-1 were compared between the Cef/Met and Ampi/Met groups (Table 2).

**Figure 2:**
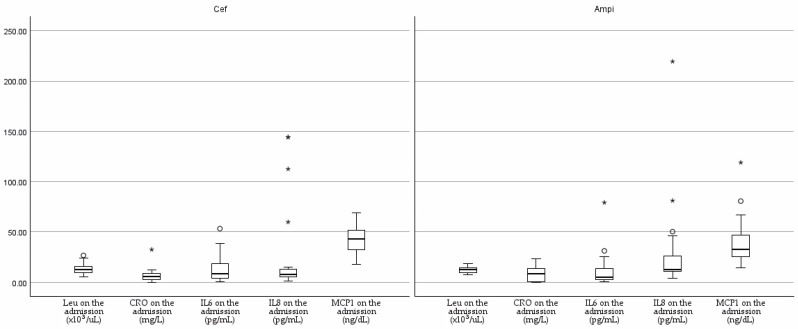
Median levels of Leu, CRP, IL6, IL8, and MCP1 on the hospital admission day (Day 1) across both antibiotic groups. Abbreviations: Starred point—extreme outliner; circle point—outliner. Levels are expressed as medians IQR (25%, 75%).

The Mann–Whitney U test was employed to evaluate the differences in inflammatory factors between the antibiotic combination groups, with each day’s median values being compared independently. On the day of hospital admission (1st day), the levels of Leu, CRP, IL-6, and MCP-1 did not vary significantly between the Cef/Met and Ampi/Met groups (Figure 2). However, a statistically significant difference was observed in the levels of IL-8 between the Cef/Met and Ampi/Met groups. Specifically, IL-8 levels were found to be significantly elevated in the Ampi/Met group (*p* = 0.034). As indicated by the analysis of inflammatory factors, no significant differences were observed between the antibiotic groups on the second and third days (for all *p* > 0.05).

Friedman’s test was utilized to assess the efficacy of treatment within a specific antibiotic group, with the objective of determining alterations in inflammatory factors. A significant decrease in both Leu and CRP was observed across both antibiotic groups after the commencement of treatment, as evidenced by a comparison of the 1st day and 3rd day values. A statistically significant difference in the distribution of Leu in the Cef/Met group was observed between the 1st and 2nd day (*p* = 0.003) and between the 1st and 3rd day (*p* < 0.001). A statistically significant difference in the Ampi/Met group distribution of Leu was identified between the 1st and 2nd day (*p* < 0.001) and the 1st and 3rd days (*p* < 0.001). Statistical analysis revealed the significant variation in the distribution of the Cef/Met CRP group between the 1st and 2nd day (*p* < 0.001) and between the 2nd and the 3rd day (*p* < 0.001). A statistically significant difference in the Ampi/Met group distribution of CRP was observed between the 2nd and the 3rd day only (*p* = 0.020).

The findings of the present study indicate a substantial decrease in IL-6 levels in the Cef/Met group. The discrepancy was observed to be statistically significant at both the 1st and 2nd day intervals (*p* = 0.002) and the 1st and 3rd day intervals (*p* = 0.003). However, no statistically significant alterations were observed in the Ampi/Met group between the 1st and 3rd days.

A comparison of the 1st admission day and 3rd revealed that other inflammatory factors, such as IL-8 and MCP-1, did not demonstrate statistically significant alterations (Table 3).

To facilitate a comprehension of the inflammatory factors’ alterations observed in both cohorts, the variation in Leu, CRP, IL-6, IL-8, and MCP-1 levels between the initial and 3rd day was calculated for each patient. This calculation entailed the subtraction of the 1st day’s and third day’s results for each individual patient.

Table 4 presents a comprehensive summary of the median difference value and Mann–Whitney U test *p*-value for each of the inflammatory factors that were the subject of analysis.

**Figure 3:**
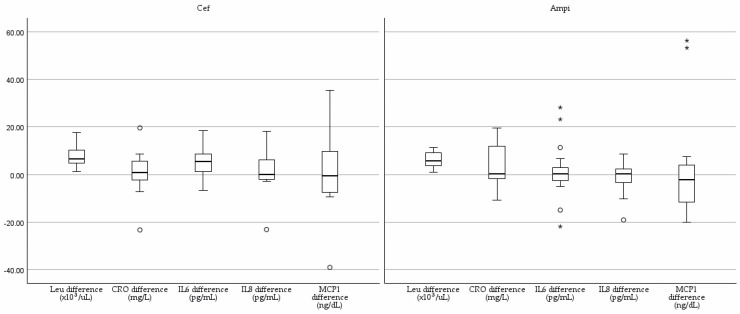
Median difference in levels of Leu, CRP, IL6, IL8, and MCP1 between 1st and 3rd hospital stay day. Abbreviations: Starred point—extreme outliner; circle point—outliner. Levels are expressed as medians IQR (25%, 75%).

In the study, levels of Leu, CRP, IL-8, and MCP-1 did not exhibit significant differences (Figure 3). However, the median reduction in IL-6 was significantly greater in the Cef/Met group than in the Ampi/Met group (*p* = 0.019), indicating a more pronounced change in IL-6 during treatment (Figure 3).

The recurrence rate was evaluated using a probable connection with a chosen antibiotic combination. The 1-year recurrence rate was 21.1% (4/19) in the Cef/Met group and 13.6% (3/22) in the Ampi/Met group, for an overall recurrence rate of 17.1% (7/41) across the cohort. However, no statistically significant association was found between antibiotic groups and appendicitis recurrence rate (OR = 0.592, 95% CI: 0.115 to 3.061, *p* = 0.685).

## 4. Discussion

The statistical analysis revealed a statistically significant decrease in Leu count in both analyzed patient groups when analyzed using Friedman’s test. Furthermore, no significance was found when comparing the median difference between the 1st and 3rd days. This finding suggests that both antibiotic groups have an equally significant impact on the decrease in Leu count.

As previously mentioned, the same can be described in relation to the CRP decrease. This decrease was found to be statistically significant in both groups when Friedman’s test was used to calculate the relevant data. The median change in C-reactive protein levels did not demonstrate statistical difference, suggesting that the decrease in CRP was even between the two groups.

During the analysis of the IL-6 group, Friedman’s test revealed a statistically significant decrease in the Cef/Met group; however, no statistically significant change was detected in the Ampi/Met group. The difference was substantiated in the analyses of median differences, where the change comparing the Cef/Met and Ampi/Met groups was found to be statistically significant, and the Cef/Met group exhibited a bigger median difference. This finding suggests the possibility that IL-6 may decrease more rapidly when the antibiotic combination of Cef/Met is used.

The IL-8 study revealed that there was neither a statistically significant change nor any significant variation in the two groups analyzed. However, it is important to note that a statistically significant difference was observed on the first day of admission to the hospital. An imbalance between the patient groups is suggested by the significant increase in the Ampi/Met group. It is possible that this demonstrates one of the limitations of the present study. Possibly, this proves the equality of antibiotic effectiveness.

The MCP-1 data revealed no statistically significant difference between the antibiotic groups. Friedman’s test did not prove statistically significant change, and the group comparison did not reveal a significant difference in the median change of the analyzed cytokine. This again suggests that both antibiotic groups are equally effective.

The odds ratio, a statistical measure of association, was calculated to describe the recurrence rate. This analysis revealed that there was no statistically significant difference between the recurrence rates of the two antibiotic groups. This finding suggests that both antibiotic groups may have equivalent efficacy in reducing the recurrence of hospitalizations.

The reduction in CRP and leukocyte count in both the target groups is consistent with the literature findings on the efficacy of antibiotics for uncomplicated appendicitis. Both leukocytes and CRP are well-established markers of systemic inflammation, with leukocytes reflecting innate immune response and neutrophil mobilization, while CRP is a protein produced by the liver after an acute inflammatory stimulus [14]. The significant reduction in both the Cef/Met group and the Ampi/Met group supports the idea that both combinations are effective in suppressing the systemic inflammatory response in children with acute uncomplicated appendicitis. This is in line with the findings from another meta-analysis of a randomized controlled trial that concluded non-operative management is safe and effective in the pediatric population, with antibiotics producing measurable improvements in inflammatory parameters [4].

The statistically significant decrease in the IL-6 observed in the Cef/Met group but not in the Ampi/Met group may be because of the broader Gram-negative antimicrobial coverage provided by ceftazidime. IL-6 is a key pro-inflammatory cytokine produced by activated macrophages, monocytes, and endothelial cells in response to infection and tissue injury and is also linked to a role in stimulating CRP [13]. In terms of appendicitis, local inflammation triggered by bacterial proliferation and luminal obstruction is associated with increased levels both locally and systemically [13,14]. IL-6 has been shown to rise earlier than CRP and more closely reflects the intensity of the inflammatory response, making it a useful diagnostic tool when used in connection with the Alvarado score [18]. Although the statistically significant difference is observed, the information should be carefully evaluated and confirmed with a larger sample population while carefully considering the inherent variability of cytokine measurements in prospective studies.

The lack of statistically significant changes in IL-8 levels within either group opens doors for further discussion. IL-8, also known as CXCL8, is the most potent chemokine involved in neutrophil activation during the acute inflammation process and is released in response mainly to bacterial infections and tissue injury [22]. High IL-8 is shown to be positively related to the intensity of chemotaxis of neutrophils towards the inflammation within the appendiceal wall [22]. Clinical studies have also shown an association between increased IL-8 and a more severe course of appendicitis [24]. The persistent elevation of IL-8 throughout the 3-day period without intra-group changes may suggest that neutrophil-mediated inflammation resolves more slowly than the systemic inflammation measured with CRP and IL-6. This reflects the sustained nature of neutrophilic activation during recovery from appendiceal inflammation and opens doors for further research on the topic. A multiplex analysis has also previously identified IL-8 as part of a panel of inflammatory markers relevant to acute appendicitis and suggested that a pattern of inflammatory markers does not normalize within a short treatment window [25]. The statistically significant difference in IL-8 levels between the two groups on the first day of admission is most likely due to the inherent heterogeneity of a small sample, rather than a true biological difference between treatment allocations, and represents an acknowledged limitation of the study.

Similarly, the absence of significant changes in MCP-1 across both groups over the study period is consistent with its role as a monocyte and macrophage chemo-attractant which is involved in a sustained rather than an acute phase of inflammation [14,16,26]. The relatively stable levels of MCP-1 across both groups indicate that the monocyte and macrophage axis of inflammatory response follows a steady course that is not substantially modulated by a short course of antibiotic therapy observed in this short time window. However, this does not diminish the overall effectiveness of both regimes since the conventional inflammatory markers of acute inflammation still stand to be CRP levels and leukocyte counts, which have significantly reduced in both groups [35].

The equivalent recurrence rates observed between the two groups align with the broader literature indicating non-operative management with antibiotics carries an acceptable risk of recurrent appendicitis. A randomized, controlled, non-inferiority trial demonstrated that non-operative management was a safe and effective management solution for pediatric patients with uncomplicated appendicitis, while acknowledging that treatment failure and recurrence rate remain important factors in clinical decision-making [7]. The current study provides additional evidence that the choice between ceftazidime/metronidazole and ampicillin/metronidazole does not significantly influence the chance of recurrence, further supporting their interchangeable use in clinical practice. As medicine moves towards individualized risk stratification rather than a uniform operative approach, the availability of multiple equivalent antibiotic options provides clinicians with greater flexibility in tailoring treatment to patient-specific factors such as allergies or local resistance patterns [10].

The role of inflammatory biomarkers in monitoring treatment response during antibiotic therapy for acute uncomplicated appendicitis remains an area of active investigation. While leukocyte count and CRP are the most widely used diagnostic non-specific markers, their low individual specificity underscores the need for a multi-marker approach [12,15]. In this context, this study provides a richer picture of the inflammatory response by using a combined trajectory of leukocyte count, CRP, IL-6, IL-8, and MCP-1 rather than just a single marker in isolation. Conventional markers do not fully showcase the complexity of the inflammatory process, which explains the interest in more specific immunological biomarkers to better characterize disease severity and therapeutic decisions [17].

In the present study, conservative treatment of acute uncomplicated appendicitis was always in line with current local pediatric guidelines, which recommend antibiotic therapy to control intra-appendiceal infection and reduce the risk of progression to perforation. The rationale for antibiotic-based conservative management is that luminal obstruction and bacterial overgrowth drive the inflammatory process; therefore, reducing bacterial load with appropriate antimicrobial coverage should dampen the inflammatory response and allow spontaneous resolution in a substantial proportion of cases.

Our experience suggests that for a certain proportion of children with uncomplicated acute appendicitis, non-surgical antibacterial therapy is an effective and safe treatment method that would avoid unnecessary surgeries due to an intact appendix, especially in combination with timely switching or conversion to surgical treatment without waiting for perforation in cases where antibiotic therapy alone does not have the desired effect.

This study has several strengths, including its randomized design, the use of a multi-marker inflammatory panel rather than a single biomarker, and 1-year recurrence follow-up. Its main limitations are the relatively small single-center sample, the absence of a formal a priori sample size calculation, and the exclusion of patients with complicated appendicitis or prior antibiotic use, which limits generalizability to the broader population presenting with suspected appendicitis.

## 5. Conclusions

Examination of the available data reveals an absence of statistically significant differences between the two antibiotic combinations. This finding suggests that both Cef/Met and Ampi/Met combinations can be utilized in clinical practice with comparable outcomes. Future multicenter studies with larger sample sizes and extended biomarker follow-up are warranted to confirm and expand upon these findings.

## Figures and Tables

**Table 1:** Characteristics of study population.

	Cef/Met	Ampi/Met	Total
Gender, n (%)			
Boy	14 (52)	13 (48)	27 (100)
Girl	5 (36)	9 (64)	14 (100)
Age, Mdn (IQR)	14 (12–15)	15 (10–16)	
Recurrence, n	4	3	7

**Table 2:** Inflammatory factors median values and Mann–Whitney U test *p*-value.

	Cef/Met, Mdn (IQR)	Ampi/Met, Mdn (IQR)	*p*-Value
Day 1			
Leu, ×10^3^/uL	12.48 (9.79–15.61)	12.51 (9.52–14.63)	0.917
CRP, mg/L	5.61 (2.46–8.66)	6.20 (0.33–13.78)	>0.999
IL-6, pg/mL	8.79 (4.07–18.44)	5.02 (2.44–13.39)	0.282
IL-8, pg/mL	7.82 (5.23–12.82)	12.69 (10.29–25.81)	0.034 *
MCP-1, pg/mL	430.38 (326.72–515.44)	324.08 (253.96–470.00)	0.158
Day 2			
Leu, ×10^3^/uL	6.59 (5.17–7.34)	6.60 (5.73–7.68)	0.861
CRP, mg/L	15.41 (6.98–25.12)	5.87 (0.69–18.53)	0.067
IL-6, pg/mL	5.38 (3.10–8.61)	5.45 (1.67–7.92)	0.819
IL-8, pg/mL	8.35 (6.40–14.61)	15.88 (8.77–29.24)	0.080
MCP-1, pg/mL	403.48 (300.35–464.23)	338.83 (293.91–495.53)	0.937
Day 3			
Leu, ×10^3^/uL	5.49 (4.54–6.29)	6.01 (4.87–7.31)	0.214
CRP, mg/L	5.03 (2.46–6.93)	1.87 (0.66–7.76)	0.062
IL-6, pg/mL	3.97 (1.32–12.38)	3.37 (2.22–15.22)	0.962
IL-8, pg/mL	7.73 (6.18–8.93)	15.00 (7.92–27.87)	0.140
MCP-1, pg/mL	389.52 (325.11–509.40)	357.54 (286.29–512.98)	0.459

* *p* < 0.05.

**Table 3:** Friedman’s test of analyzed inflammatory factors.

	Cef/Met	Ampi/Met
Leu, ×10^3^/uL	<0.001 *	<0.001 *
CRP, mg/L	<0.001 *	0.020 *
IL-6, pg/mL	0.002 *	0.756
IL-8, pg/mL	0.546	0.873
MCP-1, pg/mL	0.278	0.554

* *p* < 0.05.

**Table 4:** Median difference between 1st and 3rd hospital stay day.

	Cef/Met, Mdn (IQR)	Ampi/Met, Mdn (IQR)	*p*-Value
Leu, ×10^3^/uL	6.48 (4.59–9.96)	5.72 (3.74–9.07)	0.497
CRP, mg/L	0.48 (−2.82–5.51)	0.23 (−1.68–12.06)	0.530
IL-6, pg/mL	5.63 (1.19–8.71)	0.32 (−2.52–2.86)	0.019 *
IL-8, pg/mL	0.17 (−1.59–4.71)	0.34 (−3.28–2.29)	0.284
MCP-1, pg/mL	−1.50 (−67.52–101.34)	−20.26 (−114.58–39.83)	0.308

* *p* < 0.05.

## Data Availability

The original contributions presented in this study are included in this article. Further inquiries can be directed to the corresponding author.

## Abbreviations

The following abbreviations are used in this manuscript:

Cef/Met	ceftazidime/metronidazole
Ampi/Met	ampicillin/metronidazole
Leu	leucocyte
CRP	C-reactive protein
IL6	interleukin 6
IL8	interleukin 8
MCP1	monocyte chemoattractant protein-1
Mdn	Median
AuA	Acute uncomplicated appendicitis
